# SmartVR Pointer: Using Smartphones and Gaze Orientation for Selection and Navigation in Virtual Reality

**DOI:** 10.3390/s24165168

**Published:** 2024-08-10

**Authors:** Brianna McDonald, Qingyu Zhang, Aiur Nanzatov, Lourdes Peña-Castillo, Oscar Meruvia-Pastor

**Affiliations:** 1 Department of Computer Science, Memorial University of Newfoundland, St. John’s, NL A1C 5S7, Canada; brmcdonald@mun.ca (B.M.); lourdes.pena@mun.ca (L.P.-C.); 2 Department of Biology, Memorial University of Newfoundland, St. John’s, NL A1C 5S7, Canada

**Keywords:** smartphones and HMDs, phone in VR, virtual reality and human–computer interaction, image-based tracking, VR Interaction techniques, touch and tactile input

## Abstract

Some of the barriers preventing virtual reality (VR) from being widely adopted are the cost and unfamiliarity of VR systems. Here, we propose that in many cases, the specialized controllers shipped with most VR head-mounted displays can be replaced by a regular smartphone, cutting the cost of the system, and allowing users to interact in VR using a device they are already familiar with. To achieve this, we developed SmartVR Pointer, an approach that uses smartphones to replace the specialized controllers for two essential operations in VR: selection and navigation by teleporting. In SmartVR Pointer, a camera mounted on the head-mounted display (HMD) is tilted downwards so that it points to where the user will naturally be holding their phone in front of them. SmartVR Pointer supports three selection modalities: tracker based, gaze based, and combined/hybrid. In the tracker-based SmartVR Pointer selection, we use image-based tracking to track a QR code displayed on the phone screen and then map the phone’s position to a pointer shown within the field of view of the camera in the virtual environment. In the gaze-based selection modality, the user controls the pointer using their gaze and taps on the phone for selection. The combined technique is a hybrid between gaze-based interaction in VR and tracker-based Augmented Reality. It allows the user to control a VR pointer that looks and behaves like a mouse pointer by moving their smartphone to select objects within the virtual environment, and to interact with the selected objects using the smartphone’s touch screen. The touchscreen is used for selection and dragging. The SmartVR Pointer is simple and requires no calibration and no complex hardware assembly or disassembly. We demonstrate successful interactive applications of SmartVR Pointer in a VR environment with a demo where the user navigates in the virtual environment using teleportation points on the floor and then solves a *Tetris*-style key-and-lock challenge.

## 1. Introduction

Virtual reality (VR) typically refers to immersive graphics systems, where the user can navigate around computer-generated virtual environments and interact with 3D objects inside the environment. VR systems can be quite expensive and are usually accompanied by a set of specialized controllers held by the user in each hand and used to perform actions in the environment. Since these specialized controllers sometimes change with each new release of a VR headset, one of the barriers preventing VR from being widely adopted is the unfamiliarity that arises from all these VR controllers with different buttons and modes of operation. In this work, we show how the specialized controllers used for VR can be replaced by regular smartphones in particular situations. This would allow users to use a device they already own and are familiar with, which reduces the cost of the system and makes VR more accessible to the average consumer. Moreover, smartphones contain additional sensors and displays which can also be used to improve the interaction capabilities of regular VR systems as suggested by previous researchers [1,2,3,4,5,6].

**Figure 1:**
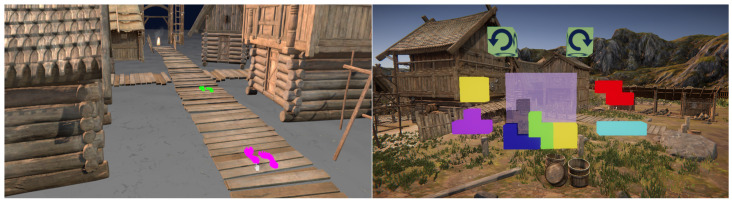
Illustration of SmartVR Pointer applications in a game-style scenario. On the (**left**), we show the navigation task within the Viking Village. Footprints are shown in bright green when they are available for selection, and turn purple indicating they will be used for teleporting there upon clicking. On the (**right**), we show the *Tetris*-style task. The *Tetris* shapes are shown in bright colors and the rotation regions are shown in green with black circles. The user drags and drops shapes to be placed in the appropriate location and orientation which is highlighted with the darker shade.

To demonstrate the feasibility of SmartVR Pointer, we implemented two sample applications. The first of these applications is a navigation task, where the user navigates around the VR environment by selecting footprint-shaped teleport markers placed on the floor using a pointer that looks like a mouse pointer, which we call a virtual pointer or simply “VR pointer”. The user can teleport by aligning the VR pointer with the footprints and tapping the phone screen to jump to the desired location. The second application illustrates a key-and-lock mechanism inspired by the video game *Tetris*. Here, the user sees a sample *Tetris*-style scene, where there are blocks and one or more empty spaces where additional blocks can fit in between the existing blocks. The user must select the correct blocks and adjust their position and orientation so that they fit inside the provided empty spaces (Figure 1). Our user study results show that the proposed technique can be used to conveniently perform common tasks in VR, such as navigation, selecting, positioning, and determining the orientation of 3D objects.

## 2. Related Work

Early work in this area explored the idea of connecting a smartphone to a VR or Augmented Reality (AR) system and allowing the user to interact with 3D objects using a combination of HMDs and Hand-Held Devices (HHDs) [7,8]. A few different approaches using a smartphone or other smart devices for interaction in VR and AR have been proposed since, with and without some type of external tracking or hardware adaptations [1,2,3,4,5,6,9,10,11,12,13,14,15]. While some approaches focus on using built-in sensors in smart devices to control hand-held ray casters or pointers [16,17,18], our approach is mainly image based, being similar in that regard to TrackCap and PAIR [4,5].

TrackCap, an image-based approach proposed by Mohr et al., involves mounting a cap on top of an HMD and using a smartphone camera to track an image printed on the underside of the cap [4]. This approach allowed them to track the position of the phone relative to the HMD and use it as an AR controller. One potential drawback of this approach is that strong back-lighting from the ceiling can cause poor visibility of the image on the cap, and users may have to compensate for this by using an additional light source underneath the cap. Since in our approach the image target is displayed on the phone screen, it does not suffer from this issue since the brightness of the phone screen can be automatically adjusted to work well under different lighting conditions.

Another alternate approach for using a smartphone as a controller for VR was proposed by Hattori and Hirai, who developed an image-based detection method based on “plane finding” [15]. This allows the phone to estimate its own position based on ambient information. However, their approach requires a specific synchronization process, and their ray-casting mechanism may fall out of alignment due to poor synchronization, whereas SmartVR Pointer does not require any form of synchronization.

Unlu and Xiao [5] proposed using a combination of a touch screen input along with internal and image-based tracking of the smartphone sensors to allow the use of the smartphone as a controller for several types of AR applications using the HoloLens. Their approach uses multiple smaller optical markers displayed around the edges of a phone screen along with built-in sensors in both the phone and HoloLens. Multiple markers are used to provide redundancy to track the phone in case any of the markers become occluded by the user’s hands during interaction. Our approach, on the other hand, implements a virtual pointer by placing a large marker at the center of the phone and casting a ray from the viewer towards the center of this marker.

Most recently, HTC released Vive Flow, a commercial smartphone-controlled VR headset aimed at the Mindfulness and Autonomous Sensory Meridian Response (ASMR) VR market [19]. It uses the phone sensors and its touchscreen to allow users to select content visible through the HMD. Vive Flow uses a wand or laser-pointer paradigm, where the wand acts as a hand-held ray caster for object selection, whereas the touchscreen is used to refine the selection or to offer various menu options. This differs from our approach in the use of optical, image-based tracking. In addition, the HTC implementation is proprietary, whereas SmartVR Pointer is distributed openly and can be downloaded here [20] under a creative commons (CC) license.

Researchers have also focused on using other smart devices such as smartwatches for interaction in VR [10,11,13,14]. One example is TickTockRay proposed by Kharlamov et al. [10], which uses the built-in sensors in a smartwatch to track the user’s arm movements and control a ray caster. Conversely, the solutions proposed by Park and Lee [14] as well as Kim and Woo [11] use hand tracking to allow users to interact with the virtual environment by performing hand gestures in front of depth sensors. A potential issue with using a smartwatch is arm fatigue caused by the user having to keep their arms raised while performing repetitive hand and wrist motions. Additionally, smartwatches are less widely adopted and less likely to be familiar to users than smartphones.

Other researchers have explored using devices that are not necessarily smart devices but contain Inertial Measurement Unit (IMU) sensors. This includes the work by Young et al. [12], where an arm-mounted input device was tested, and the work by Hincapié-Ramos et al. [9], which proposed using a smartphone-like device as a controller. These methods are prone to some degree of error accumulation caused by drift and may need re-centering or re-calibration after prolonged use.

Researchers have also proposed using smartphones and smartwatches for pointing and interacting with large 3D displays [16,17,18]. These solutions use the gyroscopes and accelerometers built in these devices to control ray casters for pointing and interacting with large displays within the VR. Like most VR controllers, they use a laser pointer metaphor, where a ray is cast from the HHD or controller towards the target or selection region. By contrast, SmartVR Pointer casts a ray from the gaze of the user (determined by the HMD camera position in world coordinates) towards the center of the QR code on the smartphone, allowing the user to refine the placement of a virtual pointer by displacing the smartphone itself.

## 3. Methods

**Figure 2:**
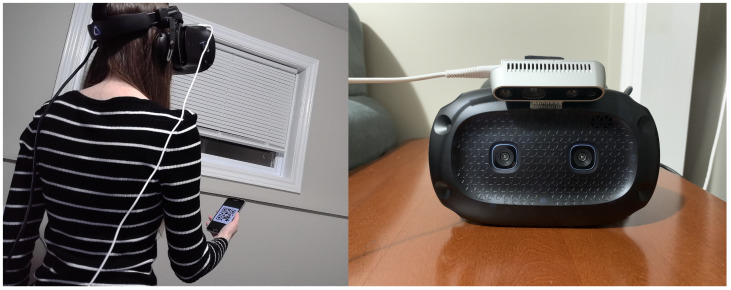
Setup for SmartVR Pointer with the camera mounted on top of the HTC Vive Cosmos Elite HMD.

**Figure 3:**
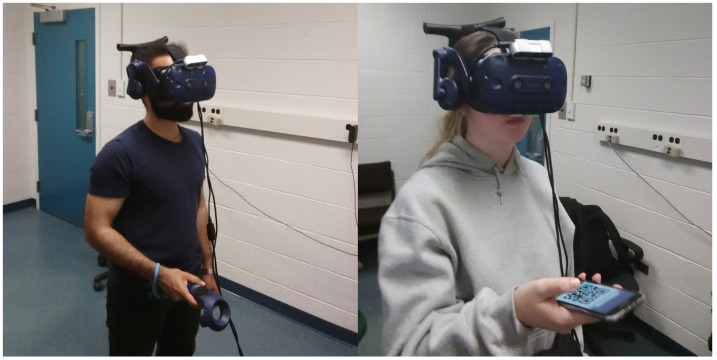
Comparison of baseline condition (**left**) to the usage of a SmartVR Pointer (**right**) with the camera mounted on top of the HTC Vive Pro Headset.

**Figure 4:**
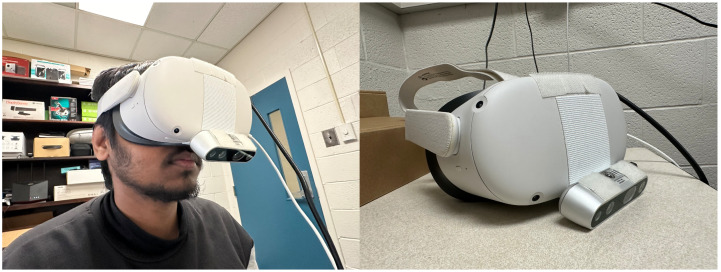
Setup for SmartVR Pointer with the camera mounted at the bottom of the Meta Quest 2 HMD.

To use SmartVR Pointer, a camera is mounted on the HMD at the height of the user’s face and tilted downwards to the region where the user would hold their smartphone comfortably, approximately at a 45 degree angle (Figure 2, Figure 3 and Figure 4). We propose three methods to define the position of the pointer used for target selection and dragging: (1) QR code tracking; (2) gaze-based tracking; and (3) a combination of both.

### 3.1. QR Tracking-Based Mode

When the selection mode is activated, which can be performed by having the user double-tap on the phone, we track a QR code displayed on the smartphone screen using the Vuforia Engine [21]. PTC Vuforia provides an AR Software Development Kit (SDK) in the form of a Unity plugin that is typically used for creating AR applications in mobile devices. Vuforia provides the functionality to easily track images and display virtual objects on top of them in AR. We utilize Vuforia’s tracking feature to track the QR code. As we track the QR code, we find out where the phone screen is located relative to the view of the camera on top of the HMD. We use this relative position of the phone to produce a pointer that the user can see inside the HMD, which is controlled by moving the smartphone within the field of view (FOV) of the camera and resembles a white mouse pointer as shown in the accompanying video.

**Figure 5:**
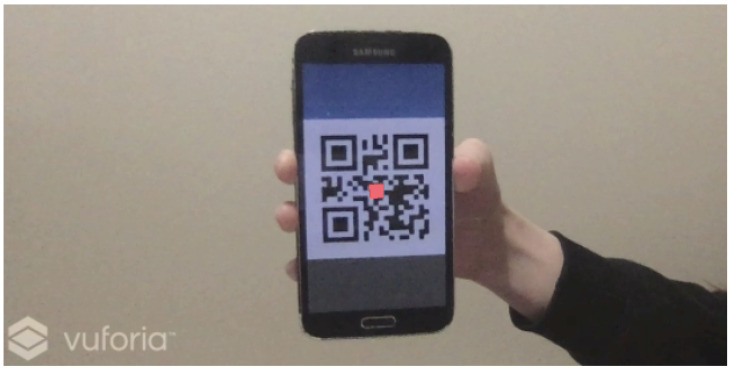
Using Vuforia Engine to display a red cube on top of the tracked QR code image displayed on the smartphone.

**Figure 6:**
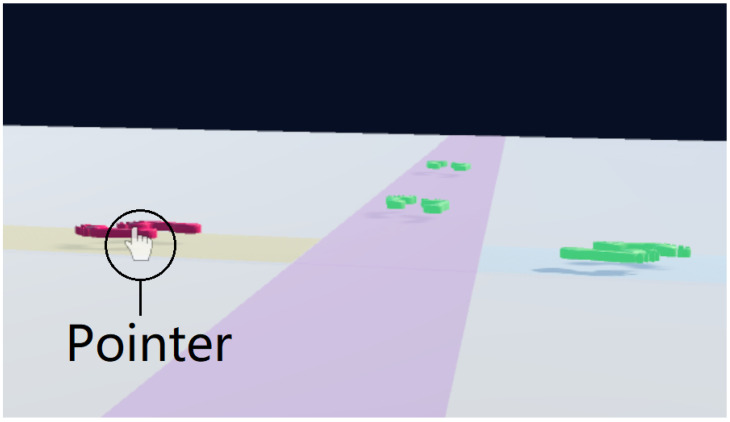
Teleporting application enabled by this technique where the user can select footprint-shaped teleport points to navigate around the VR environment by placing the pointer on one of the footprints.

**Figure 7:**
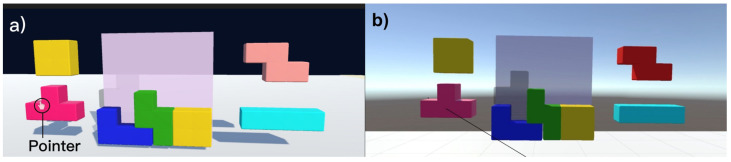
Illustration of how the ray-casting mechanism works. (**a**) The view the player sees while wearing the HMD shows a pointer highlighting the *Tetris* block in the bottom left for selection. (**b**) The developer view illustrates how a black ray is cast from the player towards the pointer, and through the *Tetris* block.

When the phone screen displaying the QR code is within the camera’s view, Vuforia is used to place a pointer on top of the center of the QR code. We can obtain the position of this pointer within the camera view (Figure 5 shows a red cube acting as a pointer). Additionally, there is a transparent 2D canvas object that covers the user’s field of view inside the VR environment and is fixed to a camera plane in front of the user. We call this the VR canvas. The position of the pointer in screen coordinates is converted to the corresponding local coordinates within the canvas. These coordinates are then used to display a pointer (similar to a desktop mouse pointer) on the canvas, which the user can see within the VR (Figure 6 and Figure 7).

At the core of this technique is a mapping process that can be decomposed into two steps: in the first step, the Vuforia engine returns the coordinates of the QR code with respect to the real world camera as an X, Y, Z vector. We discard the Z coordinate (which is Vuforia’s estimate of depth) and use the X, Y coordinates as the coordinates of the tracked object within the AR camera plane, which is a 2D plane that does not correspond to any location in the VR. We use the X, Y coordinates and the size of the AR camera plane to calculate the position of a pointer as a proportion of the width and height of the AR camera. These coordinates provide the relative displacement of the QR code within the field of view of the AR camera and are used in the second step. In step 2, we use the relative position of the tracked object to determine the actual position of the VR pointer within the VR canvas. This is performed by determining the positions of the corners of the VR canvas in world coordinates, which vary according to the position and orientation of the HMD. Since we know the relative proportion of width and height of the tracked object in the AR camera, and we have the corners of the canvas in world coordinates, we can calculate the corresponding position of the VR pointer in world coordinates. This way, the VR pointer is an object that always lies within the VR canvas. The mechanism behind the tracking transforms a world space point (position of the red cube in Figure 5) into a position in the local space of a rectangular pointer canvas (in the VR) using Unity’s ScreenPointToLocalPointInRectangle() method and is presented in Algorithm 1: QR code Mapping to VR canvas.
**Algorithm 1** QR code mapping to VR canvas.**Input:** InputQR[x,y] coordinates of a QR code tracked by Vuforia.**Output:** Position[x,y]                ▹ Pointer position on canvas let n be a top left corner position[x,y] of a QR image let m be a bottom right corner position[x,y] of a QR image **if** QR code IS tracked by Vuforia **then**      let minX, minY, maxX, maxY be the 4 boundaries of the pointer canvas      newPos = Unity.ScreenPointToLocalPointInRectangle(n,m,InputQR,canvas);             ▹ Mapping from QR coords to a point in the VR canvas      **if** (minX≤newPos.X≤maxX) and (minY≤newPos.Y≤maxX) **then**          position[x,y] = [newPos.X,newPos.Y] ▹ Updates pointer position on the canvas      **end if** **else if** QR code IS NOT tracked by Vuforia **then**      position[x,y] = [(maxX+minX)/2,(maxY+minY)/2] ▹ Pointer is set to canvas center **end if**

As a result of this mapping process, the VR pointer is controlled by moving the phone and can be used as a pointer for interaction. This pointer function is then used for creating a raycast from the user towards the pointer location on the canvas. The raycast extends indefinitely to hit objects in the background (Figure 7). It is invisible to the user and simply allows us to detect which 3D object is in line with the pointer and the user’s point of view at any given time.

Lastly, there is a wireless transfer of data between the smartphone and the VR application running on a desktop computer. This allows the smartphone to communicate with the VR application and permits the user to perform various actions in VR by interacting with the phone screen. In particular, we can send a message to the VR application when the user presses their finger against the phone screen or releases their finger from it. We use this functionality to implement various types of clicking and hold-and-release interactions that are commonly used in VR. The proposed setup has the advantage that the system provides tactile/haptic feedback to the users, increasing their confidence during selection tasks. Touch is an important feature in systems that use smartphones in VR, as it has been shown that direct touch provides faster and more precise interactions [22].

### 3.2. Gaze-Based Mode

**Figure 8:**
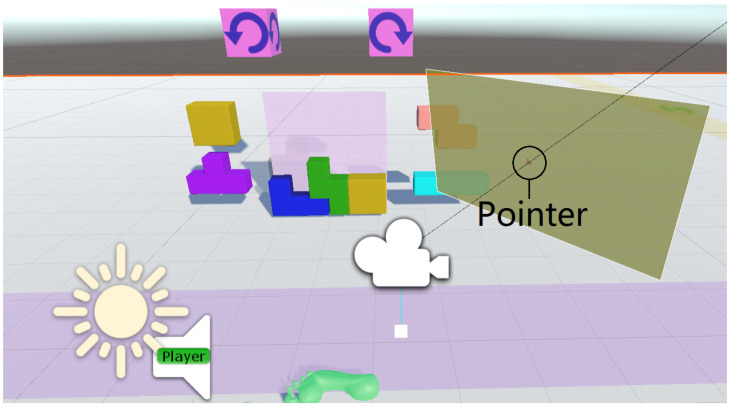
Screenshot of the *Tetris*-like task view shown in Unity from a third-person perspective, illustrating the semi-transparent VR canvas (the rectangle shown on the right side, darkened here for emphasis). From the user’s perspective (the white camera in the middle), the panel appears fixed in front of the viewer.

In this mode, a red mouse pointer appears in the center of the FOV of the user wearing the HMD. Users then move their head to move the pointer around the scene. As the pointer is moved over eligible target locations, those locations become highlighted, indicating they are potential selection targets. The user then taps on the smartphone screen to confirm the target selection. In the case of a drag-and-drop operation, the object remains selected until the user stops touching the smartphone screen. Figure 8 illustrates, from a 3rd person perspective, how this VR panel is attached to the HMD view and how a ray is cast from the camera to the pointer.

### 3.3. Combined Mode

The combined mode uses both methods alternately. It defaults to QR-based tracking when the QR code is visible from the point of view of the camera and shows a white mouse pointer while the tracking is active. If either the QR code is not found within the FOV of the user, or the Vuforia plugin is unable to track the smartphone for any reason, the system turns into head-gaze-based selection [23], and the mouse pointer turns red to indicate the gaze-based mode is active. Then, the user adjusts the direction of their head to control the position of the red pointer on the 2D canvas and select either a footprint for navigation or the *Tetris*-like pieces to solve a puzzle. The selection and release of *Tetris*-like pieces is performed by the user pressing the smartphone’s touchscreen to initiate a drag operation and later dropping an item by ending the contact with the touchscreen. If at any point the QR code becomes visible again, the VR pointer turns white and the user can refine its positioning by moving the mouse around in front of the viewer.

### 3.4. Advantages of SmartVR Pointer

An advantage of SmartVR Pointer is that it only requires a regular camera mounted on top of the HMD. Potentially, the built-in cameras of some HMDs could also be used, with the drawback that the user may need to hold the smartphone a bit higher than in our proposed configuration, potentially increasing arm fatigue. An alternative configuration for the system is to set up a wireless camera (e.g., a GoPro), which could stream to the desktop app running the main application. This removes the need for the extra cable to the webcam but inserts network delay that comes from the wireless transmission of the camera frames, making the method slow to react to the user’s actions. In our current setup, we use an Intel RealSense Depth-Sensing camera because this camera can be connected to longer USB cables and provides depth video for other applications. For the demo applications, we implement our solution on two HMDs: a Vive Cosmos (Figure 2) and a Meta Quest 2 (Figure 4). In both cases, we use the RealSense camera as a regular RGB camera.

Another advantage is that the solution we present does not need calibration. Slight deviations of the camera from the suggested position, which might be caused by removing it and mounting it back in place, would not be an issue as long as the camera is fixed with respect to the HMD during operation. As the user will be guided by the position of the virtual pointer within the VR environment analogous to the use of a mouse pointer in the desktop metaphor, similar to when a user finds a regular mouse pointer by moving the mouse around and finding it on the screen as the mouse pointer responds, a SmartVR user will find the pointer in the VR as long as there is detection of the QR code in the smartphone, or a red pointer in the gaze-based mode appears, and then refine the positioning to the desired target. To point to a particular location using QR-code based tracking, the user naturally moves the smartphone to the left, right, up, or down of the virtual panel in front of them, and that will translate in the virtual pointer moving accordingly. When QR tracking is turned off or unavailable, we use gaze-based positioning to establish the selection target, and the user then presses the smartphone screen to indicate a selection, or to start a drag-and-drop sequence.

Additionally, users can regulate the angle of the camera with respect to the HMD. As mentioned above, if the camera used for tracking faces straight towards the front, the user will need to raise their hands higher up or move their gaze towards the floor. If the camera faced directly towards the floor, the user would have to move the phone on a quasi-horizontal plane (perpendicular to the camera). We have found that when the camera faces about 45 degrees down from the line of sight (or front axis) towards the vertical axis (for a user standing in an upright position), it is possible for the user to comfortably hold the phone, looking at a plane that is tilted roughly 45 degrees with respect to the upright position, which is typical when using the smartphone.

### 3.5. Availability, Reproducibility, and Setup of SmartVR Pointer

The sample applications for this project were implemented using the Unity 3D game engine. The entire Unity project for SmartVR Pointer, including its source code is freely available by accessing the link provided here [20], which also contains installation instructions PDF S1 (Supplementary Materials) and troubleshooting tips.

In order to reproduce SmartVR Pointer, a user needs the Unity engine with the Vuforia plugin installed on their computer, an HMD, a webcam with a form factor and weight suitable for attaching it to the HMD, and a velcro strip with adhesives to attach the webcam to the HMD. Alternatively, a built-in camera in the HMD could be used if it points roughly towards the region where users hold their smartphones and the HMD SDK allows access to the camera’s video stream. SmartVR Pointer does not require any calibration, but the camera needs to point roughly towards the general region where the user wants to hold their smartphone in a comfortable way. Once the camera is set up, the camera needs to be registered in the Unity project as per the installation instructions.

## 4. Sample Applications

The first application we implemented involves allowing the player to navigate around a virtual environment (Figure 6). The user is presented with multiple paths that have 3D footprints at various points along them that represent teleport points. To select a teleport point, the user first lines up the pointer with one of the footprints and taps the smartphone screen. This will teleport the player by moving them to the location of the footprint and updating their rotation to match the direction that the selected footprint is facing.

The second application we implemented is a key-and-lock mechanism based on the video game *Tetris* (Figure 7). In this demo, the user is presented with a *Tetris* puzzle with an empty goal space represented by a semi-transparent gray block showing where another block can fit. To complete the task, the user must select, position, and rotate the correct block into the correct position in the puzzle in a drag-and-drop fashion. The user can perform this by selecting the correct block by aligning the pointer with it and then pressing and holding their finger against the phone screen until releasing at the right spot and in the orientation that matches the *Tetris*-like challenge. This causes the block to move with the pointer so the user can drag it into position by moving the smartphone. The user can also rotate the block by swiping the phone screen with their thumb by 25 degrees to the left or right, and this will rotate the block 90 degrees in the direction that they are swiping. Alternatively, users can rotate the item by moving it within the region of a clockwise rotation widget that is shown on top of the *Tetris*-like challenge. Each time the dragged object aligns with the rotation icon, the item is rotated accordingly.

## 5. User Study

We performed a user study with 30 participants using a game-style scenario to find out if the performance of SmartVR Pointer users is comparable to that of users of commercial VR controllers for the applications discussed in Section 4. Participants were recruited through MUN email distribution lists and postings in social media. Ethical approval was received from the Interdisciplinary Committee on Ethics in Human Research (ICEHR), Memorial University of Newfoundland, and study participants were required to complete an ethics consent form. Figure 1 illustrates a model of a Viking Village provided by Unity, which we employed in our study. After one trial round, each participant performed 10 measured rounds moving around the Viking Village under three different conditions: The first was using a regular VR controller (the baseline condition) followed by either the gaze-based SmartVR Pointer selection, the tracker-based SmartVR Pointer selection, or the combined/hybrid mode. Participants were randomly assigned to one of the conditions with a pre-test confidence test and post-tests of the perception of the ease of use and interaction mode preference. The user’s performance was assessed based on the completion time and click success rate.

### 5.1. Statistical Analysis

To test whether the composition of the three condition groups with respect to gender, occupation, age, HMD experience, HMD frequency of use, and handedness was as expected from a random participant group allocation, we performed Pearson’s Chi-square tests with *p*-values calculated by Monte Carlo simulation with 10,000 replicates. To test whether the participants’ pre-test confidence was similar among the three condition groups, we performed Wilcoxon tests with false discovery rate (FDR) correction for multiple testing rounds. To test whether participants’ responses to Likert-scale questions in the post-test questionnaire differed among condition groups, we performed pair-wise Wilcoxon tests with false discovery rate (FDR) correction for multiple testing rounds. ANOVA (type II) analyses were performed to assess the effect of condition, gender, round, and age on the mean completion time and mean click success rate. All statistical data analyses were conducted in R (version 4.1.3). Plots were created using R data visualization package ggplot2 (version 3.4.3). ANOVA analyses were performed using the Anova function available in the car R package (version 3.1-2).

### 5.2. Results

**Figure 9:**
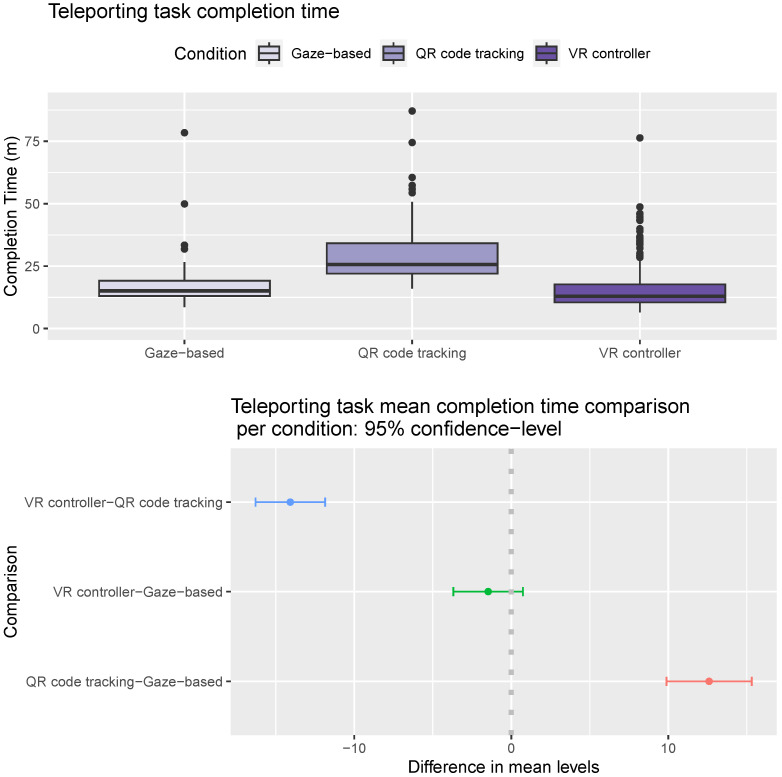
Teleporting task completion times. (**Top**) Distribution of completion time per condition. Horizontal line inside the box indicates the median completion time, and the box height indicates the interquartile range (IQR). (**Bottom**) Pairwise mean time completion difference between conditions. The gray dotted line marks the point where the difference between the mean levels of the conditions compared is zero.

Results from an ANOVA analysis suggest that the most significant factors in the teleporting task completion time of the participants are the condition (F statistic 117.13, *p*-value < $2\times 10^{-16}$) and the round (F statistic 18.82, *p*-value < $2\times 10^{-16}$). Gender is a moderately significant factor (F statistic 7.58, *p*-value 0.006), and age is not a significant factor (F statistic 1.52, *p*-value 0.22). Male participants were slightly faster in this task than female participants (mean completion time 18.04 m vs. 19.89 m, Tukey multiple comparisons of means *p*-value 0.014). Users completed the teleporting task faster in later rounds suggesting a learning effect. As shown in Figure 9-Top, users using the gaze-based approach and VR controller had significantly lower completion times in the teleporting task than users using the QR code-tracking approach (Tukey multiple comparisons of means *p*-value < $1\times 10^{-7}$). Users were slightly faster using the VR controller than the gaze-based approach (mean completion time 17.1 m vs. 15.63 m, Tukey multiple comparisons of means *p*-value 0.26, Figure 9-Bottom).

**Figure 10:**
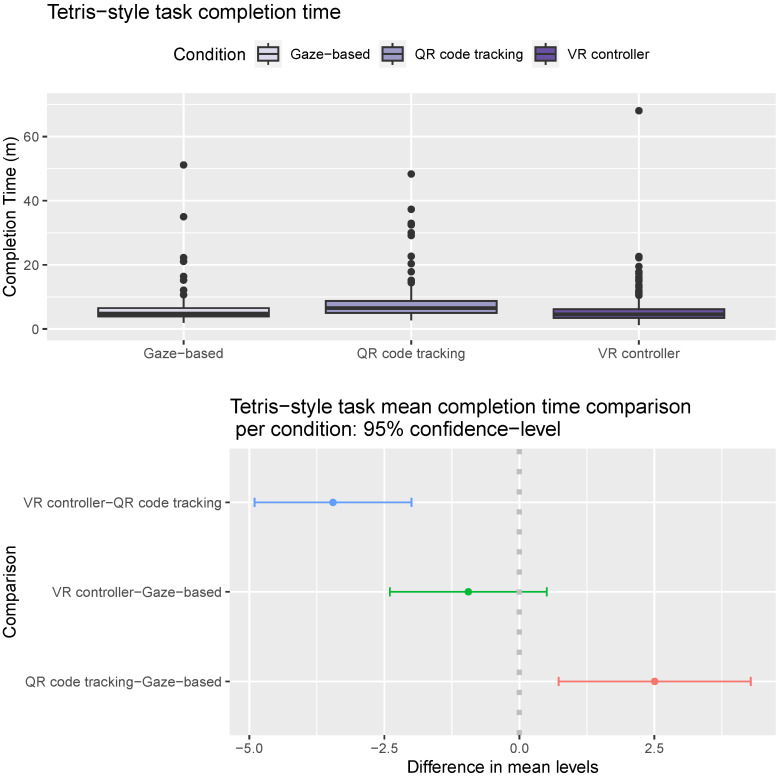
*Tetris*-like task completion times. (**Top**) Distribution of completion time per condition. Horizontal line inside the box indicates the median completion time, and the box height indicates the interquartile range (IQR). (**Bottom**) Pairwise mean time completion difference between conditions. The gray dotted line marks the point where the difference between the mean levels of the conditions compared is zero.

The ANOVA analysis results suggest that the most significant factors in the *Tetris*-like task completion time of the participants are again the condition (F statistic 17.84, *p*-value < $2\times 10^{-16}$), and the round (F statistic 8.26, *p*-value $1.79\times 10^{-11}$). Gender is also a significant factor (F statistic 15.20, *p*-value 0.0001), and age is a moderately significant factor (F statistic 4.51, *p*-value 0.012). Male participants were slightly faster in this task than female participants (mean completion time 5.73 m vs. 7.33 m, Tukey multiple comparisons of means *p*-value 0.001). Participants in the age range 24–48 were slightly slower in this task than participants in the age range 18–24 (mean completion time 6.91 m vs. 5.48 m, Tukey multiple comparisons of means *p*-value 0.012). As shown in Figure 10 (Top), users using the gaze-based approach and VR controller had significantly faster completion times in this task than users using the QR code-tracking approach (Tukey multiple comparisons of means *p*-values 0.003 and $1\times 10^{-7}$). Users were slightly faster using the VR controller than the gaze-based approach (mean completion time 6.41 m vs. 5.46 m, Tukey multiple comparisons of means *p*-value 0.28, Figure 10-Bottom).

**Figure 11:**
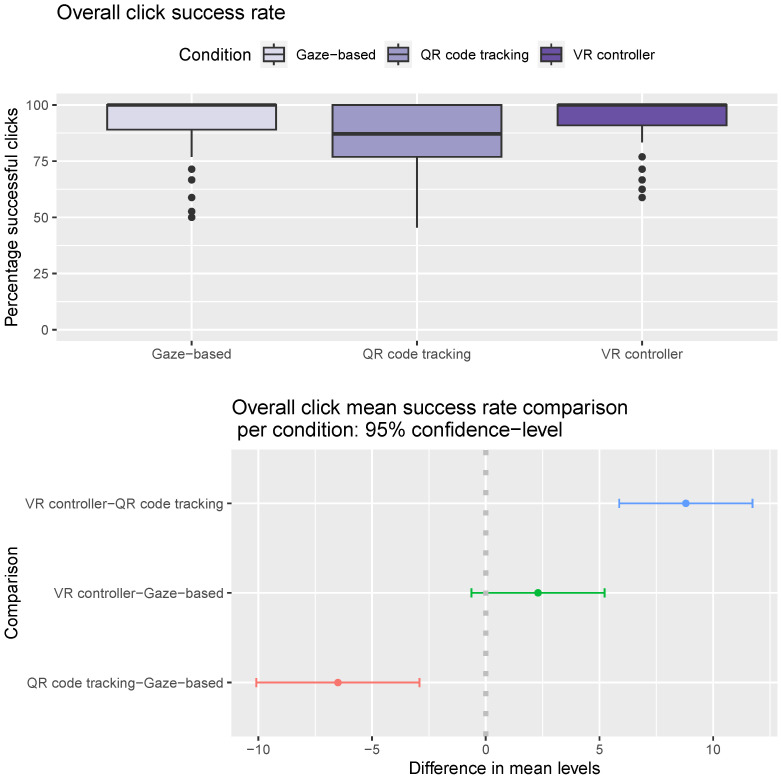
Click success rate. (**Top**) Distribution of click success rate per condition. Horizontal line inside the box indicates the median click success rate, and the box height indicates the interquartile range (IQR). (**Bottom**) Pairwise mean click success rate difference between conditions. The gray dotted line marks the point where the difference between the mean levels of the conditions compared is zero.

We calculated the click success rate of the participants as the percentage of successful clicks (successful clicks divided by total number of clicks). Based on the ANOVA results, the most significant factor in the click success rate of the participants is the condition (F statistic 24.72, *p*-value $5.99\times 10^{-11}$). All other factors (gender, age, and round) were not statistically significant. Participants have a higher click success rate using the gaze-based approach and VR controller than when using the QR code-tracking approach (Tukey multiple comparisons of mean *p*-values 0.00007 and <$1\times 10^{-7}$, Figure 11).

**Figure 12:**
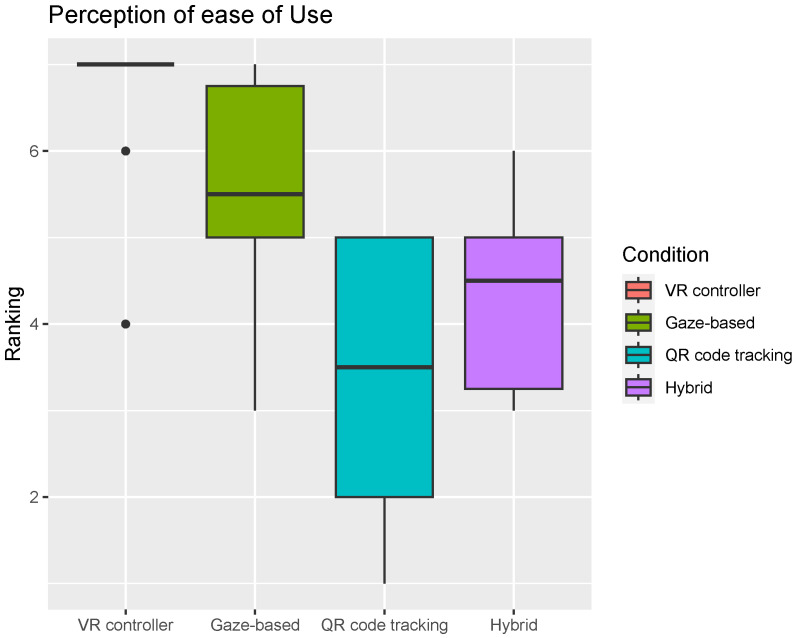
Participants scores regarding perception of ease of use per condition. Horizontal line inside the box indicates the median click success rate, and the box height indicates the interquartile range (IQR). For Ranking, 1 is “Least Preferred” and 7 is “Most preferred”.

Participants perceived the VR controller to be easier to use than any of the other three conditions (Wilcoxon-adjusted *p*-values 0.001, $9.1\times 10^{-7}$, and $9.1\times 10^{-7}$ for gaze-based, QR code-tracking and hybrid condition, respectively). Participants also perceived the gaze-based approach to be easier to use than the QR code-tracking and hybrid mode (Wilcoxon-adjusted *p*-values 0.009 and 0.047). There was no statistically significant difference in the users’ perception of ease of use between the QR code-tracking and hybrid condition (Figure 12).

**Figure 13:**
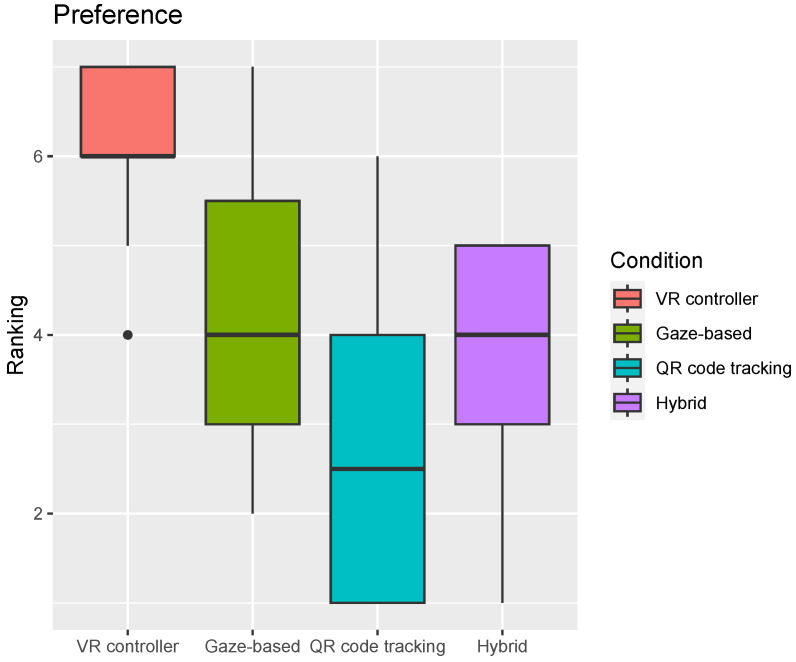
Participants scores regarding condition preference. Horizontal line inside the box indicates the median click success rate, and the box height indicates the interquartile range (IQR). For Ranking, 1 is “Least Preferred” and 7 is “Most preferred”.

Participants reported preferring to use the VR controller to solve both tasks more than any of the other three conditions (Wilcoxon-adjusted *p*-values 0.003, $1.6\times 10^{-5}$, and $1.6\times 10^{-5}$ for gaze-based, QR code-tracking and hybrid condition, respectively). Users also reported preferring to use the gaze-based condition over the QR code-tracking condition (Figure 13).

**Figure 14:**
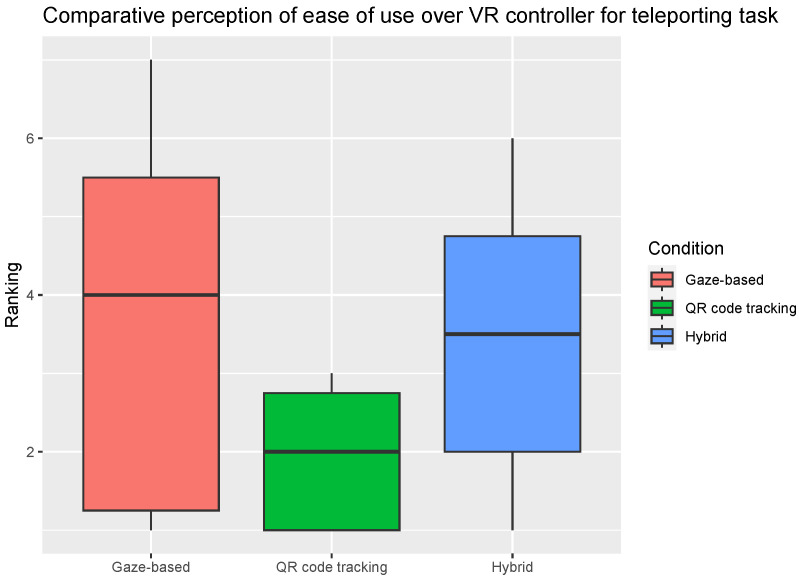
Participants agreement with the statement that one of the three SmartVR Pointer modes was easier to use for the teleporting task than the VR controller. Horizontal line inside the box indicates the median click success rate, and the box height indicates the interquartile range (IQR). For Ranking, 1 is “Not agree at all” and 7 is “Completely agree”.

**Figure 15:**
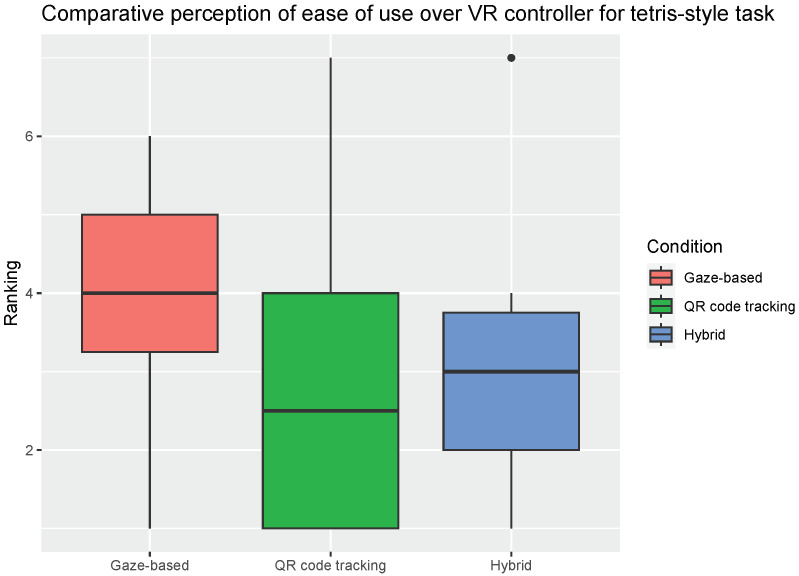
Participants agreement with the statement that one of the three SmartVR Pointer modes was easier to use for the *Tetris*-like task than the VR controller. Horizontal line inside the box indicates the median click success rate, and the box height indicates the interquartile range (IQR). For Ranking, 1 is “Not agree at all” and 7 is “Completely agree”.

When participants were asked to agree or disagree on whether one of the SmartVR Pointer method was easier to use than the VR Controller for each task, there was no statistically significant difference between the conditions (Figure 14 and Figure 15). However, the median score (4 for both tasks) given to the gaze-based condition was higher than the median score given to the other two conditions (Figure 14 and Figure 15). This suggests that the users felt neutral about replacing the VR controller with the gaze-based condition instead of reluctant as with the other two conditions.

#### Unstructured Feedback Results

Supporting the results from the statistical analysis, the overall feedback from participants on their experience during the experiment and overall outcome of them completing the challenges and navigating around the virtual scene was a slight preference for VR controller usage (baseline condition) compared to the other modes (QR code tracking, gaze based, and combined). This might be due to the users being already familiar with the VR controller, as 15 of the participants reported to have previous experience using a VR controller.

The gaze-based approach proved to be a worthy contestant to the VR controller, as the gaze-based mode was the participants’ second choice regarding the ease of navigation and selection during the experiment. In total, 50% of the participants did not express any significant preference towards the usage of any of the methods, although the combined mode was slightly (about 5%) more preferred than the other approaches. The participants liked the idea of the QR tracking-based mode (10% more frequently preferred and picked it as the best method compared to other methods); despite finding it more difficult to learn and get used to, they performed better and better at every experiment round.

Overall, 46.62% of population remained ‘neutral’ on picking the best option and not expressing any preference towards one of the options provided for the navigation or selection tasks exposed to them, with 20.00% preferring to use the VR controller and 17.25% picking one of the proposed navigation methods as the favored ones. Also, 16.13% of the participants expressed a negative impression towards one of the navigation methods provided to them.

## 6. Discussion

One of the main implications of our findings is that gaze-based SmartVR Pointer can be used as an alternative to the use of VR controllers under certain circumstances, such as when users do not have access to the VR controllers that are usually delivered with the HMDs, or when these are simply unavailable. For instance, SmartVR Pointer could be used in conjunction with the Apple Vision Pro or Microsoft’s HoloLens, which are HMDs that do not come with VR controllers by default. Another implication is that, as the users’ performance in terms of task completion time and click success rate was comparable between the VR controller and the gaze-based mode, the gaze-based SmartVR Pointer method should be the primary choice when using SmartVR Pointer. Furthermore, the gaze-based mode was the users’ second best rated condition in terms of perception of ease of use and interaction mode preference.

### 6.1. Limitations

A limitation of the QR code tracking approach is the fact that, as in any optical-based sensing system, if there are objects blocking the line of sight between the smartphone and the HMD, the QR code is not readable and tracking will be lost. This is a common limitation for many of the related research we have mentioned, and even optical trackers of actual VR controllers must remain visible to the base stations or optical sensors. For instance, tracking will be lost if a finger or a hand is placed between the phone’s camera and the QR code in the TrackCap system [4]. When placing the QR code, we made sure that it was highly visible and large, and left room on the sides to allow users to hold the phone and tap the screen for interaction. By doing this, the tapping operation did not interfere with the phone tracking. A different proposal to address this issue is presented in the PAIR system, where an array of smaller QR codes is placed around the screen of the smartphone, ensuring one of the codes can be tracked at any time [5].

A limitation of the gaze-based mode is that users are required to center their view in their selection by actively positioning their head accordingly, whereas in the QR code tracking system, users have the possibility to keep their head positioning stable or semi-static while moving the phone in front of them to displace the pointer to the desired target position.

In terms of prolonged use, our findings and experience suggest that the quality and mechanism of the HMD head-strapping mechanism has an influence on the degree of comfort of the users in the long term. For instance, the default straps provided in the Meta Quest 2 seem to be less comfortable over prolonged use than the premium straps that come with the HTC Vive Pro or the HoloLens. The latest HMDs come with premium strap options that are more comfortable to wear and easier to adjust than the elastic straps from previous generations. We suggest their regular use to increase comfort and improve the practical viability of our approach.

A final limitation is that users are not able to directly see the smartphone screen inside the VR. This could be problematic under some circumstances, such as when the user is trying to pick up the phone from a table or a desk. To counter this, some HMDs allow the rotation of the HMD towards the ceiling, allowing users to find their smartphones while temporarily searching for it. Another alternative to see the smartphone could be to use an Augmented Reality HMD, such as the Meta Quest 3 or the Apple Vision Pro, provided the corresponding SDK gives developers access to the built-in front camera video stream, or to use Near-Range Augmented Virtuality, as proposed by Alaee et al. [1], to allow VR users to see the smartphone and other items in close proximity.

### 6.2. Future Work

Several researchers have proposed to use smartphones in Augmented Reality (AR) setups. We believe that SmartVR Pointer would be ideal for see-through/AR HMDs. When wearing a see-through HMD, such as Microsoft’s HoloLens or the Magic Leap, a user would be able to see the smartphone directly and use the virtual pointer to select objects within the Augmented Reality environment, similar to what is shown in Figure 5 and in the supporting video. The viability of such an approach was shown by Unlu et al., who used the HoloLens’ front camera in the PAIR system [5].

Several new HMDs that are not see-through, such as the Meta Quest 3 and the Apple Vision Pro, have recently been commercialized and come with built-in cameras to provide a pass-through mode that allows users to see their real world surroundings. A proposed improvement for SmartVR is to use the built-in cameras in non-see-through HMDs, which would eliminate the need for an external camera as well as the possibility of the camera being accidentally detached or disconnected. A related challenge with some HMDs, such as the HTC Vice Cosmos, is that the built-in cameras point towards the front, which forces users to either raise the phone higher than in the proposed setup, or to look down to find the smartphone. More recently, some HMDs, such as the Valve Index, Apple Vision Pro, and the Meta Quest 3, have positioned their pass-through cameras to look halfway down to capture the users’ hands and surroundings without forcing the users to raise their hands in front of them. These pass-through cameras use ultra-wide-angle lenses (also known as fish-eye lenses), which cause severe perspective distortions in the captured frames. These distortions make it very challenging for the Vuforia engine to track the QR code, as the plugin is designed to process images from cameras with regular lenses and views. An optical tracking system designed to compensate for such wide-angle lens distortions could be used to track the QR code in these HMDs. A potential problem with this approach is that currently, not all HMD manufacturers provide game engine developers with SDK access to the video streams from the pass-through cameras.

Finally, it is worth noting that the Vuforia plugin provides a measure of the tracked target’s depth so that it could be possible to assess the approximate distance from the user’s viewpoint to the phone. When the user moves closer to or away from the camera, a 3D pointer could use this depth information. Depending on the application, it might be useful to access the depth information for spatial tasks such as 3D sculpting. Such 3D positioning would require that the distances detected by the Vuforia plugin be mapped to distances in the virtual environment. In this work, we found that keeping the pointer on a 2D projection plane in front of the user provides a robust and intuitive solution for selection and interaction with virtual objects, and that the depth channel information might introduce a confounding factor for the operations we have implemented.

We are planning to study additional essential operations in Extended Reality (XR) that can be performed as quickly and efficiently as when using a custom VR controller. We are also interested in finding out whether more of the functions afforded by current VR controllers can be replaced by smartphones and look forward to developing additional applications of this technique for virtual, mixed, and Augmented Reality.

### 6.3. Conclusions

We have presented SmartVR Pointer, a technique that allows the use of the smartphone, a device already in use by most people, for intuitive, one-handed tactile interaction in VR that eliminates the need for specialized controllers for the demonstrated applications. The proposed approach offers the following advantages:

1. It allows for the use of the smartphone in virtual, mixed and Augmented Reality and supports the sense of touch for object selection, dragging and navigation.
2. It eliminates the need to learn how to use custom VR controllers for completing these tasks.
3. It works with a regular camera and is compatible with HMDs that already come with a front camera.
4. It is simple to set up and requires no calibration, synchronization, or complex hardware assembly or disassembly.

The approach we have described has the potential to make VR more accessible to the average consumer through a reduction both in the learning curve associated with using most VR controllers and in the need for specialized controllers for VR systems. Through the application demos, we found that SmartVR Pointer can be reliably used for navigation as well as for selection, positioning, and rotation of 3D objects within VR. This is promising, as many interactions in VR consist of some combination of these basic tasks.

## Data Availability

The Project download for SmartVR Pointer is available online at https://drive.google.com/drive/folders/1aZy3rgfaQnb1dKR1UosWUP0r5ryAKLiw?usp=sharing.

## Supplementary Materials

The following supporting information can be downloaded at https://www.mdpi.com/article/10.3390/s24165168/s1: PDF S1: Installation instructions.
