# Supplementary material for: SmartVR Pointer: Using Smartphones and Gaze Orientation for Selection and Navigation in Virtual Reality
Source: Sensors (Basel). 2024 Aug 10;24(16):5168. doi: 10.3390/s24165168 (PMC11359486; doi:10.3390/s24165168)
Supplement: Supplementary file 1 [file sensors-24-05168-s001.zip › sensors-3042218-supplementary.pdf]

PDF S1

Instructions for Installing and Running the Project

How to Install and Set Up the Server Unity Project:

- Requirements:
  - Unity version 2020.3.25f.
  - SteamVR.
  - VIVE software, or the equivalent software for another VR headset if you are not using a VIVE system.
- Download the VRServer.zip file and extract its contents.
- Open the Unity Hub and click on the "Open" button.
- Select the folder where you extracted the project. The project should be added to the Unity Hub and open automatically in the Unity Editor.
- Select the Player game object from the Hierarchy on the left side of the screen (Figure S1).

- Use the Move Tool to drag the Player game object in front of the demo you want to try. The Rotate Tool can also be used to adjust the direction the Player is facing when the application starts.

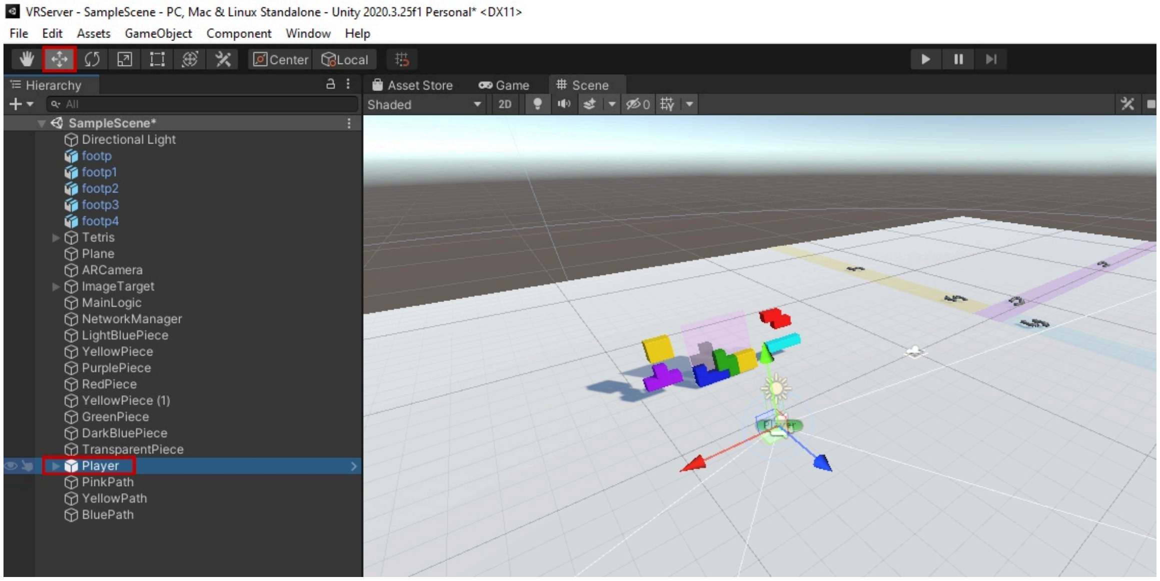

Figure S1: An image showing how to edit the Player's starting position in the Unity Editor.

How to Install and Set Up the Client Unity Project:

- Requirements:
  - Unity version 2020.3.25f.
  - An Android smartphone. This application was tested using a Samsung Galaxy S5 but will likely work with any newer Android smartphone.
- Download the VRClient.zip file and extract its contents.

- Open the Unity Hub and click on the "Open" button.
- Select the folder where you extracted the project. The project should be added to the Unity Hub and open automatically in the Unity Editor.
- Select the NetworkManager game object from the Hierarchy on the left side of the screen (Figure S2).
- In the Inspector on the right side of the screen, under the "Network Manager (Script)" section, edit the "Ip" field value to be the internal IP address of the host computer (Figure S2). The IP address can be found by running the "ipconfig" command using Command Prompt.
- Click on "File" and then "Build Settings..." in the top bar of the Unity Editor.
- Make sure Android is selected as the platform, then click the "Build" button and save the created .apk file.
- The .apk file created in the last step is the Android app. This file must be transferred to the Android smartphone and installed. One easy way to do this is by uploading the .apk file to Google Drive on your computer and then opening Google Drive on the smartphone and tapping on the file to download it.
- Open the VRClient app once it has finished installing.
- When the desktop VR application is running, tap the QR code on the smartphone screen to connect the smartphone to the server.

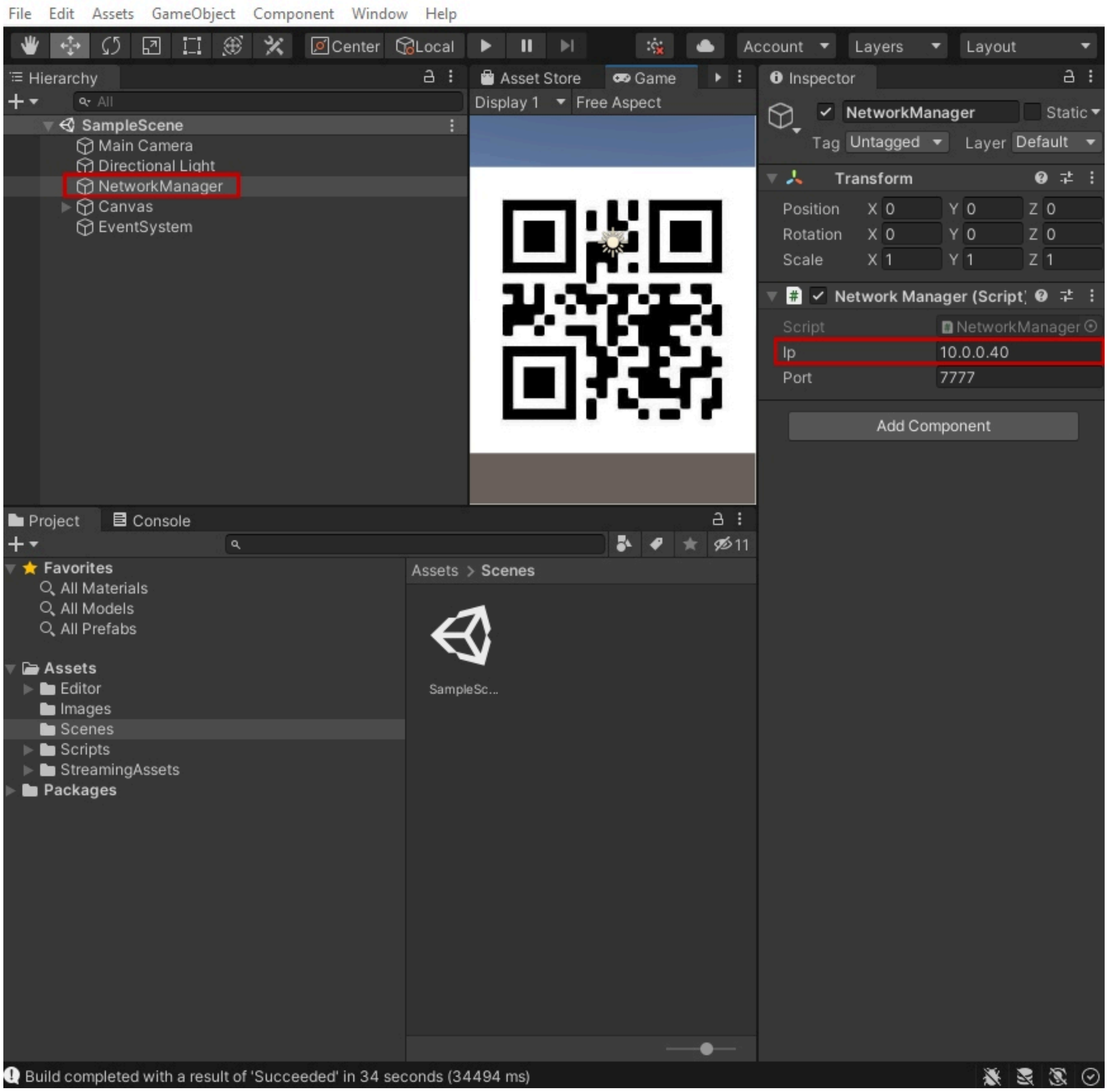

Figure S2: An image showing where to edit the IP address in the VRClient project.

Solutions to Potential Issues:

- Network Connection Between Smartphone and Computer Not Working:
  - Make sure the IP address entered for the NetworkManager game object in VRClient is correct.

    - Check your firewall settings to make sure it is not blocking IP address connections.

- Issues Receiving Video Stream from Camera:

    - Search for the ”webcamprofiles.xml file” within the VRServer project folder and open it.
    - Add the information for the camera you are using to the ”webcamprofiles.xml” file.
    - Open the VRServer project in the Unity Editor.
    - Select the ARCamera game object from the Hierarchy on the left side of the screen.
    - In the Inspector on the right side of the screen, under the ”Vuforia Behaviour (Script)” section, click on the ”Open Vuforia Engine configuration” button.
    - Under the ”Play Mode” section of the Vuforia Configuration, select the camera you are using in the ”Camera Device” dropdown menu.

- Pointer is Too High or Low:

    - Certain setups may require a manual adjustment to make the pointer easier to use.
    - Find the Canvas game object under Player → SteamVRObjects → VRCameraParent → VRCamera → Canvas and select it.
    - Move the Canvas object slightly up or down using the Move Tool until the pointer is working better.
